# Medulloblastoma or not? Crucial role in tumorigenesis of the timing of migration of cerebellar granule precursor cells, regulated by Nos2 and Tis21

**DOI:** 10.3389/fnins.2012.00198

**Published:** 2013-01-24

**Authors:** Stefano Farioli-Vecchioli, Laura Micheli, Luca Leonardi, Manuela Ceccarelli, Sebastiano Cavallaro, Felice Tirone

**Affiliations:** ^1^Institute of Cell Biology and Neurobiology, National Research Council, Fondazione Santa LuciaRome, Italy; ^2^Functional Genomics Center, Institute of Neurological Sciences, National Research CouncilCatania, Italy

**A commentary on**

**Nos2 inactivation promotes the development of medulloblastoma in Ptch1(+/−) mice by deregulation of Gap43-dependent granule cell precursor migration**

by Haag, D., Zipper, P., Westrich, V., Karra, D., Pfleger, K., Toedt, G., Blond, F., Delhomme, N., Hahn, M., Reifenberger, J., Reifenberger, G., and Lichter, P. (2012) PLoS Genet. 8:e1002572. doi: 10.1371/journal.pgen.1002572

**Tis21 knock-out enhances the frequency of medulloblastoma in Patched1 heterozygous mice by inhibiting the Cxcl3-dependent migration of cerebellar neurons**

by Farioli-Vecchioli, S., Cinà, I., Ceccarelli, M., Micheli, L., Leonardi, L., Ciotti, M. T., De Bardi, M., Di Rocco, C., Pallini, R., Cavallaro, S., and Tirone, F. (2012) J. Neurosci. 32, 15547–15564.

Medulloblastoma is a very aggressive tumor of the cerebellum and one of the most common malignant pediatric brain tumors. Medulloblastoma comprises four tumor subtypes; about one fourth of medulloblastomas originate from precursor cells of granule neurons (GCPs), carrying an aberrant activation of the Sonic Hedgehog proliferative signaling (Shh; Yang et al., 2008; Gibson et al., 2010).

It is in fact accepted that prolonged mitotic activity of GCPs at the surface of the cerebellum during its postnatal morphogenesis makes the cells potential targets of transforming insults (Wang and Zoghbi, 2001).

Two recent studies (Farioli-Vecchioli et al., 2012a; Haag et al., 2012) have highlighted that the localization of preneoplastic GCPs (pGCPs) during cerebellar development plays a crucial role for their malignant progression. These studies show that ablation of Nos2 (nitric oxide synthase) or of Tis21 (also known as Btg2 or PC3) leads to impairment of the migration of GCPs from the surface of the cerebellum toward the internal layers. This occurs in consequence of the decrease of expression of two genes regulated by Nos2 and Tis21, i.e., Gap43 and the chemokine Cxcl3, respectively, which specifically induce GCPs migration. Ablation of either Nos2 or Tis21 in Shh-activated mice leads to a large increase in the frequency of medulloblastoma. The explanation for such an increase, supported by data, is that the prolongation of the permanence in the external proliferative cerebellar region under control of Shh exponentially increases the possibility of neoplastic transformation. In the study of Farioli-Vecchioli et al. (2012a) the specificity of the effect of Cxcl3 on the migration of GCPs is guaranteed by the observations that Cxcl3 cell-autonomously regulates their migration without affecting either proliferation or differentiation. Furthermore, ablation of Tis21 does not influence the proliferation of GCPs [also suggesting that other genes of the same family expressed in the cerebellum, such as Btg1 (Farioli-Vecchioli et al., 2012b), may vicariate the known antiproliferative action of Tis21]. Although the ablation of Tis21 reduces the differentiation of GCPs (Farioli-Vecchioli et al., 2012a), it is known that GCPs exit the cell cycle and start differentiating after migrating away from the surface of cerebellum (Choi et al., 2005).

Some additional considerations arise from the Farioli-Vecchioli et al. (2012a) study. First, Canzoniere et al. (2004) have previously proposed that Tis21 overexpression induces differentiation of GCPs by inducing Math1, a gene known to support the differentiation of GCPs (Gazit et al., 2004). Consistently, the ablation of Tis21 causes the down-regulation of Math1 in cerebellar precursors (Figure A1A). This, however, appears to conflict with recent reports indicating that Math1 is required for the formation of medulloblastomas induced by constitutive activation of the Shh pathway (Zhao et al., 2008; Flora et al., 2009). Moreover, Math1 behaves as a tumor suppressor in colorectal cancer (Bossuyt et al., 2009). An interesting possibility reconciling these observations was proposed by Flora et al. (2009). When cerebellar precursors are in a proliferative environment Math1 makes the cells competent to transduce the proliferative signal of Shh. In contrast, when the cells are exposed to a differentiative signal, Math1 has a prodifferentiative action. In keeping with this idea, it is possible that the ablation of the prodifferentiative gene Tis21 in Shh-activated mice, depriving the GCPs of a differentiative stimulus, would favor the pro-Shh action of Math1. Consequently, the action of activated Shh on GCPs at the surface of the cerebellum would became more penetrant. A putative model of the Math1 pathway in GCPs (relative to Tis21) is illustrated in Figure 1A (based on the above references and on: Hammerle and Tejedor, 2007; Farioli-Vecchioli et al., 2009); Figure A1A shows the expression values in Tis21-knockout GCPs of Math1 and of Id3, an inhibitor of proneural genes which is a direct target of Tis21 in the dentate gyrus and in GCPs (Farioli-Vecchioli et al., 2009 and data not shown).

**Figure 1:**
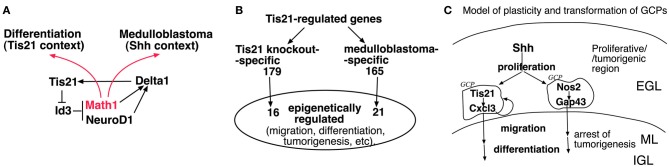
**Proposed models of plasticity and transformation of cerebellar granule precursor cells (GCPs) in response to Math1, Tis21, Nos2, Cxcl3, or Gap43. (A)** Hypothetical model for the ambivalent role of Math1 (Atoh1) in tumorigenesis and differentiation; in a proliferative (Shh) environment Math1 favors tumorigenesis, while when GCPs are exposed to a differentiative signal (Tis21), Math1 may have a prodifferentiative action. Math1 and Id3 were not selected among the 344 genes whose expression is modified by Tis21-knockout in the report by Farioli-Vecchioli et al. (2012a), because of the stringent cut-off imposed on the statistical analysis. See Figure A1 for analysis of Math 1 and Id3 mRNA levels in GCPs of Tis21-null mice. **(B)** Epigenetically-regulated genes among the 344 Tis21-regulated genes, either Tis21-knockout-specific (pairwise comparison of *Tis21*^*KO*^ vs. *Tis21*^*WT*^) or medulloblastoma-specific (pairwise comparison of *Ptch1*^+/−^/*Tis21*^*KO*^ vs. *Ptch1*^+/−^/*Tis21*^*WT*^); see (Farioli-Vecchioli et al., 2012a) and Figure A1 for enrichment analysis. **(C)** Model of plasticity and transformation of GCPs, involving Cxcl3- and Gap43-induced migration (see text). EGL, external granular layer (surface of cerebellum); ML, molecular layer; IGL, internal granular layer.

Secondly, Tis21 is a transcriptional cofactor, known to be recruited as part of protein complexes containing histone modifying factors to which Tis21 is known to bind, namely, the protein-arginine N-methyltransferase PRMT1, and the histone deacetylases HDAC1 and HDAC4 (Lin et al., 1996; Passeri et al., 2006; Farioli-Vecchioli et al., 2007). These complexes may control the activity of multiple transcription factors by epigenetic mechanisms and account for at least part of the changes in gene expression observed. Such a possibility was tested by interrogating the array of 344 Tis21-regulated genes, either Tis21-knockout-specific or medulloblastoma-specific (Farioli-Vecchioli et al., 2012a), in a search for Tis21-dependent genes targets of epigenetic modifiers. Indeed, a significant number of genes turn out to be epigenetically modifiable, either being responsive to histone deacetylase inhibitors, or because their products bind histone deacetylase proteins. Some of these genes are involved in cell migration, contraction or motility, or in tumorigenesis (see Figures 1B, A1B).

This suggests that the transcriptional control exerted by Tis21 on cohorts of genes involved in the neoplastic transformation of GCPs may occur at least in part epigenetically. It would be interesting to verify whether the same occurs for Nos2.

A more general and important question raised by these studies concerns the possibility of controlling the development of medulloblastoma by regulating the migration of GCPs. Farioli-Vecchioli et al. (2012a) show that exogenous Cxcl3 can reduce the area of medulloblastoma lesions in cerebellar slices. It is known that pGCPs can still differentiate and migrate like normal GCPs, although they are able to generate a tumor when transplanted (Kessler et al., 2009). Thus, it is possible to speculate that the migration-promoting action of Cxcl3 or Gap43 may induce pGCPs to differentiate and exit the neoplastic program. Nonetheless, it is plausible that, after a yet undefined period of time, pGCPs may become irreversibly transformed and lose the ability to differentiate. If so, it is necessary that the treatment with proteins regulating GCPs migration such as Cxcl3 or Gap43 takes place at the very initial stages of the tumor (see model in Figure 1C). Otherwise, the induction of migration at later stages may contribute to cancer cell spreading, in which case it would be more appropriate to inhibit migration, using for instance the chemokine Cxcl12, which keeps GCPs at the surface of cerebellum, and is upregulated in Tis21-null GCPs (Tiveron and Cremer, 2008; Farioli-Vecchioli et al., 2012a). These considerations highlight the need for routine checks, with more powerful techniques to diagnose brain tumors at a very early stage.

## Figures and Tables

**Figure A1:**
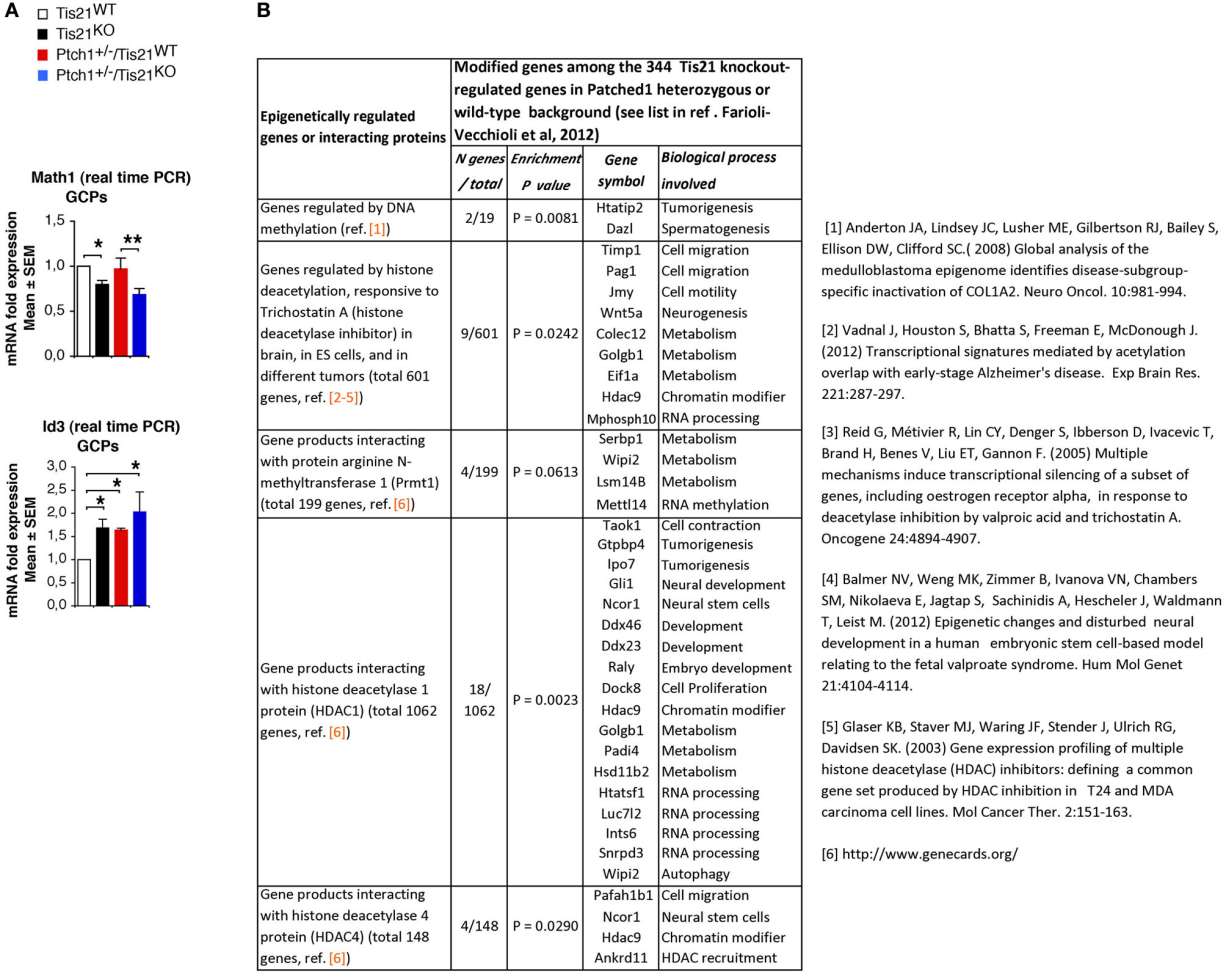
**(A)** Math1 and Id3 mRNA expression values by real time PCR analysis performed in GCPs from 7-days-old mice of the four genotypes indicated. Math1 mRNA levels are reduced in GCPs of Tis21 knockout mice, either in a Patched1 wild-type background (*Tis21*^*KO*^ vs. *Tis21*^*WT*^) or in a Patched1 heterozygous background (*Ptch1*^+/−^/*Tis21*^*KO*^ vs. *Ptch1*^+/−^/Tis21^*WT*^). Mean ± SEM fold increases are from three independent experiments. TBP was used to normalize data. ^*^P < 0.05, or ^**^P < 0.01, Student's *t*-test. **(B)** Enrichment analysis to identify epigenetically-regulated genes among the Tis21-regulated genes. Among the 344 Tis21-regulated genes, either Tis21-knockout-specific (pairwise comparison of *Tis21*^*KO*^ vs. *Tis21*^*WT*^) or medulloblastoma-specific (pairwise comparison of *Ptch1*^+/−^/*Tis21*^*KO*^ vs. *Ptch1*^+/−^/*Tis21*^*WT*^; see Farioli-Vecchioli et al., 2012a) genes were selected whose product interacts with histone modifying factors [i.e., arginine N-methyltransferase 1 (Prmt1), or histone deacetylases] or whose expression is significantly affected by inhibitors of DNA methylases or histone deacetylases. Enrichment *P* values were obtained with Fisher's exact test.
